# Musculoskeletal Development and Skeletal Pathophysiology’s

**DOI:** 10.3390/ijms23169092

**Published:** 2022-08-13

**Authors:** Elizabeth W. Bradley

**Affiliations:** Department of Orthopedics, Stem Cell Institute, School of Medicine, University of Minnesota, Minneapolis, MN 55455, USA; ebradle1@umn.edu

Musculoskeletal (MSK) disorders are one of the leading causes of disability for people of all ages and impart significant socio-economic burdens on society [1,2]. MSK disorders are the leading cause of disability in people under 65-years-old, and the second leading cause thereafter [1]. These disorders represent more than 50% of disabling health conditions reported by adults [1]. Two major forms of musculoskeletal disease include osteoarthritis, characterized by degradation of articular cartilage within synovial joints, and osteoporosis, typified by reduced skeletal bone mass and increased risk of fracture. Moreover, muscle wasting and tendon/ligament injuries further expand the burden of MSK disorders. Understanding the disease mechanisms and underlying principles of how these tissues are formed and maintained throughout the lifespan is critical to our ability to thwart MSK injury and degeneration. This Special Issue includes literature reviews, as well as basic science reports, within these areas of musculoskeletal research to broaden our understanding of MSK tissue development and degeneration.

Osteoarthritis is a disease of the entire joint. This includes synovial fibroblasts, articular cartilage chondrocytes, underlying cells within the subchondral bone, as well as invading vasculature, neurons and hematopoietic cells within the joint. Understanding how each of these cell types contribute to OA will help design therapies to mitigate disease progression. Two manuscripts within this Special Issue characterize the synoviocyte secretome and its impact on cartilage destruction. Vasoactive intestinal peptide (VIP) is a hormone that has broad effects, including immunomodulatory properties. Prior studies demonstrated that VIP can prevent joint destruction and limit inflammation within OA joints [3], but how VIP impacts inflammatory mediator production in human OA joint cells was unknown. Using stable isotope labeling with amino acids (SILAC), Perez-Garcia et al. documented VIP-mediated regulation of 115 secreted proteins produced by co-cultured human synovial fibroblasts and articular chondrocytes, and showed that VIP limits the expression of inflammatory mediators by human joint cells [4]. In addition to inflammatory mediators, synoviocytes also produce small extracellular vesicles (EV) that contain nucleic acids, proteins, lipids and metabolites. Mustonen et al. show that human synovial fibroblasts produce EVs containing hyaluronic acid [5]. Moreover, they show that membrane fluidity, governed by the composition of fatty acids with the EV lipid bilayer, changes with cell proliferation in vitro that is accompanied by diminished EV and HA production [5].

Cathelicidin-related antimicrobial peptide (Cramp) is an antimicrobial molecule produced by innate immune cells that functions to promote cell proliferation and migration. In work by Choi et al., the authors demonstrate that inflammatory mediators induce Cramp expression by chondrocytes and that Cramp stimulates chondrocyte catabolism, and accelerates OA progression in a murine model [6]. Ramesova et al. demonstrate that caspase-1 helps to regulate chondrocyte differentiation and lipid metabolism, suggesting a potential role for caspase-1 in diet-induced osteoarthritis, in which increased levels of systemic inflammation are observed [7]. These studies support that limiting inflammation within the joint may have beneficial effects on cartilage preservation.

Inflammation and innate immune cells likewise promote bone loss. Bone mass in the adult skeleton is controlled through the balanced actions of bone-resorbing osteoclasts and bone-forming osteoblasts. In addition, osteocytes and hematopoietic cells within the bone marrow also influence this process. Numerous conditions, including pregnancy, lactation, advanced age and glucocorticoid use, can alter the balance of bone formation to bone resorption, resulting in net changes to bone mass. In this Special Issue, Tomaszewska et al. utilize spiny mice (*Acomys cahirinus*) that have a longer gestational period and fewer pups per litter to better mimic the effects of pregnancy on the body in humans [8]. The authors show that supplementation with β-hydroxy-β-methylbutyrate during the second trimester prevents bone loss during pregnancy [8]. While pregnancy enhances bone resorption, women are also more prone to osteoporosis. In a manuscript by Karkache et al., the authors expand upon prior work, showing that the protein phosphatase Phlpp1 limits bone mass in females [9]. The authors demonstrate that genetic ablation of Phlpp1 within myeloid progenitors enhances bone mass in females by limiting bone resorption [9]. Women exhibit a marked increase in bone loss following the menopause. In work by Nagao et al., the authors show that cytotoxic T lymphocyte-associated antigen-4Ig (CTLA-4Ig) limited bone resorption and mechanical hyperalgesia in a model of post-menopausal bone loss in mice (e.g., ovariectomy) [10].

Inflammatory bone loss can also occur in response to wear particles derived from orthopedic implants (e.g., fixation devices). Magnesium alloys simulate the mechanical properties of bone and have low toxicity, but the high degradation rate in vivo elicits an inflammatory response. Negrescu et al. show that coating a magnesium alloy with electrospun poly(ε-caprolactone) fibers, loaded with coumarin and/or zinc nanoparticles, reduced the degradation rate of magnesium alloys [11]. Furthermore, they demonstrate that coated magnesium alloys also limit osteoclast differentiation in vitro, as compared to uncoated controls, suggesting that coated magnesium implants may reduce the inflammatory response [11].

Therapeutic use of glucocorticoids attenuates inflammation, but also has detrimental side effects on the musculoskeletal system. This includes loss of bone and muscle mass. Work by Webster et al. shows that global loss of the enzyme 11β-hydroxysteroid dehydrogenase type 1 (11β-Hsd1) protects against glucocorticoid-induced muscle wasting in a murine model of polyarthritis (e.g., TNFα transgenic mice). The authors also demonstrate enhanced 11β-HSD1 activity within muscle biopsies of rheumatoid arthritis patients that correlates with inflammatory markers [12]. 

In addition to these important basic research manuscripts, this Special Issue also includes pertinent literature reviews within the field of musculoskeletal development and skeletal pathophysiologies. Our hope is that this work provides some insight into the ongoing efforts to prevent and treat musculoskeletal diseases.

## References

[1] boneandhealthburden.org. The Burden of Musculoskeletal Diseases. https://www.boneandjointburden.org/.

[2] Cosman F., De Beur S.J., LeBoff M.S., Lewiecki E.M., Tanner B., Randall S., Lindsay R. (2022). The clinician’s guide to prevention and treatment of osteoporosis. Osteoporos Int..

[3] Jiang W., Wang H., Li Y.-S., Luo W. (2016). Role of vasoa.active intestinal peptide in osteoarthritis. J. Biomed. Sci..

[4] Pérez-García S., Calamia V., Hermida-Gómez T., Gutiérrez-Cañas I., Carrión M., Villanueva-Romero R., Castro D., Martínez C., Juarranz Y., Blanco F. (2021). Proteomic Analysis of Synovial Fibroblasts and Articular Chondrocytes Co-Cultures Reveals Valuable VIP-Modulated Inflammatory and Degradative Proteins in Osteoarthritis. Int. J. Mol. Sci..

[5] Mustonen A.-M., Paakkonen T., Matilainen J., Rilla K., Käkelä R., Malinen M., Takabe P., Oikari S., Capra J., Sihvo S.P. (2022). Fatty Acid Fingerprints and Hyaluronic Acid in Extracellular Vesicles from Proliferating Human Fibroblast-like Synoviocytes. Int. J. Mol. Sci..

[6] Choi M.-C., Jo J., Lee M., Park J., Park Y. (2021). Intra-Articular Administration of Cramp into Mouse Knee Joint Exacerbates Experimental Osteoarthritis Progression. Int. J. Mol. Sci..

[7] Ramesova A., Vesela B., Svandova E., Lesot H., Matalova E. (2021). Caspase-1 Inhibition Impacts the Formation of Chondrogenic Nodules, and the Expression of Markers Related to Osteogenic Differentiation and Lipid Metabolism. Int. J. Mol. Sci.

[8] Tomaszewska E., Donaldson J., Kosiński J., Dobrowolski P., Tomczyk-Warunek A., Hułas-Stasiak M., Lamorski K., Laskowska-Woźniak D., Muszyński S., Blicharski R. (2021). beta-Hydroxy-beta-Methylbutyrate (HMB) Supplementation Prevents Bone Loss during Pregnancy-Novel Evidence from a Spiny Mouse (*Acomys cahirinus*) Model. Int. J. Mol. Sci..

[9] Karkache I.Y., Damodaran J.R., Molstad D.H.H., Mansky K.C., Bradley E.W. (2021). Myeloid Lineage Ablation of Phlpp1 Regulates M-CSF Signaling and Tempers Bone Resorption in Female Mice. Int. J. Mol. Sci..

[10] Nagao N., Wakabayashi H., Miyamura G., Kato S., Naito Y., Sudo A. (2020). CTLA-4Ig Improves Hyperalgesia in a Mouse Model of Osteoporosis. Int. J. Mol. Sci..

[11] Negrescu A.M., Necula M.-G., Gebaur A., Golgovici F., Nica C., Curti F., Iovu H., Costache M., Cimpean A. (2021). In Vitro Macrophage Immunomodulation by Poly(epsilon-caprolactone) Based-Coated AZ31 Mg Alloy. Int. J. Mol. Sci..

[12] Webster J.M., Sagmeister M.S., Fenton C.G., Seabright A.P., Lai Y.-C., Jones S.W., Filer A.F., Cooper M.S., Lavery G.G., Raza K. (2021). Global Deletion of 11beta-HSD1 Prevents Muscle Wasting Associated with Glucocorticoid Therapy in Polyarthritis. Int. J. Mol. Sci..

